# Intrathoracic Caecal Perforation Presenting as Dyspnea

**DOI:** 10.1155/2010/296730

**Published:** 2011-01-20

**Authors:** Vincent Granier, Emmanuel Coche, Philippe Hantson, Maximilien Thoma

**Affiliations:** ^1^Service des Soins Intensifs, Cliniques Saint-Luc, Université Catholique de Louvain, Avenue Hippocrate 10 1200, Brussels, Belgium; ^2^Service de Radiologie, Cliniques Saint-Luc, Université Catholique de Louvain, Avenue Hippocrate 10 1200, Brussels, Belgium; ^3^Service de Chirurgie et Transplantation Abdominale et service des Urgences, Cliniques Saint-Luc, Université Catholique de Louvain, Avenue Hippocrate 10 1200, Brussels, Belgium

## Abstract

*Introduction*. Bochdalek hernia is a congenital defect of the diaphragm that is usually diagnosed in the neonatal period and incidentally in asymptomatic adults. Small bowel incarceration in a right-sided Bochdalek hernia is exceptional for an adult. *Case Presentation*. A 54-year-old woman was admitted for acute dyspnea, tachycardia, hypotension, and fever. Five days before, she had been experiencing an episode of diffuse abdominal pain. The admission chest X-ray was interpreted as right pleural effusion and pneumothorax with left mediastinal shift. Chest tube drainage was purulent. The thoracoabdominal CT examination suspected an intestinal incarceration through a right diaphragmatic defect. At laparotomy, a right-sided Bochdalek hernia was confirmed with a complete necrosis of the incarcerated caecum. Ileocaecal resection was performed, but the patient died from delayed septic complications. *Conclusion*. Intrathoracic perforation of the caecum is a rare occurrence; delayed diagnosis due to misleading initial symptoms may lead to severe complications and poor prognosis.

## 1. Introduction

Faecopneumothorax is a very rare but potentially fatal occurrence due to the perforation of the colon or the small bowel into the thorax. It may occur as a complication of a traumatic diaphragmatic injury but is exceptional after a congenital defect. We describe an unusual presentation of a Bochdalek hernia in an adult complicated by a right-sided incarceration and secondary perforation of the caecum.

## 2. Case Report

**Figure 1:**
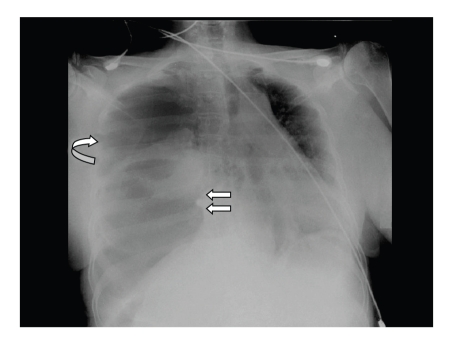
Bedside chest radiograph showed a right upper lucency (curved arrow) with left mediastinal shift in relationship with tension pneumothorax. The air present in the lower part of the right hemithorax was retrospectively interpreted as caecal distension with perforation.

A 54-year-old woman presented to the Emergency Department for acute dyspnea. She had a past medical history of rheumatoid arthritis and for many years was treated by steroids and etanercept. There was no history of previous abdominal trauma. Five days earlier, while staying abroad for holidays, she went through diffuse abdominal pain including diarrhoea and vomiting. The patient had local medical consultation, where physical examination was found unremarkable. An abdominal ultrasound was reported as normal; no laboratory tests nor chest X-ray examination were deemed necessary. She was prescribed nonsteroidal anti-inflammatory drugs for pain relief. The abdominal pain progressively disappeared, but anorexia persisted. During the flight back, 5 days later, the patient complained from acute dyspnea. On arrival in the Emergency Department, the patient was tachypneic (respiratory rate > 40/min) and presented peripheral cyanosis. The pulse oxygen saturation (SpO_2_) on room air was only 75%. The other vital signs were temperature 38.4°C, heart rate 133/min, and arterial blood pressure 97/36 mmHg. Lung auscultation revealed diminished breathing sounds in the right field. The abdomen was soft with normal bowel sounds. Laboratory investigations revealed elevated C-reactive protein (CRP) 44.6 mg/dL (<1), white blood cell count 10200/mm³, blood urea 136 mg/dL (15–50), and serum creatinine 1.55 mg/dL (0.6–1.4). Arterial blood gas analysis showed an arterial pH at 7.38, with PCO_2_ 26 mmHg, total bicarbonate 15 mmol/L, and lactate 3 mmol/L. The admission chest X-ray showed a large lucent right hemithorax with left mediastinal shift (Figure 1). The patient was suspected to have tension pneumothorax.

An anterior chest tube was placed and drained 500 mL of pus. Microscopic examination of the pleural fluid revealed the presence of at least three microorganisms. Admission blood cultures were positive for *Clostridium tertium* and *Bacteroides fragilis*. 

**Figure 2:**
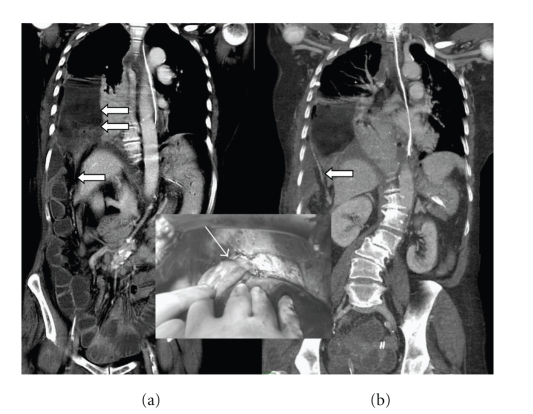
Multidetector CT performed after contrast medium injection and reformation in the frontal plane (a) revealed a large defect in the right diaphragm with herniation of the right colon (straight arrow). Fluid present above the right diaphragm is explained by the distension and perforation of the caecum (double arrows). Note the dilatation of the small bowel loops in relationship with incarceration of the caecum through the diaphragm defect. Maximal intensity projection (MIP) performed on a posterior plane (b) revealed the migration of the right colonic artery (straight arrow) with the ascension of the right colon. Inset: intraoperative view of the right colon herniated through the Bochdalek hernia (arrow).

Since the patient did not have a typical history for a lung infection and her initial symptoms were abdominal pain, a contrast-enhanced thoracoabdominal computed tomography (CT) was performed (Figure 2). This examination showed a large right pneumothorax with lung collapse and an air-fluid collection in place of the lower right pulmonary lobe. Small bowel distension was noted with a flat transverse and left colon. A small bowel obstruction due to incarceration through a right diaphragmatic defect was suspected.

Exploratory laparotomy was performed and showed an incarcerated right diaphragmatic hernia containing the terminal ileum and the caecum up to the middle part of the right colon (Figure 2). The diaphragmatic defect was enlarged radially, and the completely necrotic and perforated caecum was reduced into the abdomen. An ileocaecal resection was performed with terminal ileostomy and mucus fistula on the colon. Transdiaphragmatic pleural lavage and right lung decortication was performed. After placement of two large-bore chest tubes and reexpansion of the right lung, the diaphragmatic defect was sutured. 

The clinical condition improved rapidly after surgery. However, on the 6th postoperative day, a new thoracoabdominal CT was done due to the recurrence of fever with a high suspicion of sepsis. It showed multiple right chest wall abscesses that required surgical debridement and drainage. Resistant *Enterococcus faecalis* was identified in the abscesses and *Pseudomonas aeruginosa* in blood cultures. Unfortunately, the patient also developed necrotizing fasciitis of the thighs and lower abdomen which led to refractory septic shock and finally death.

## 3. Discussion

Faecopneumothorax is a rare occurrence that usually results from the perforation of the bowel after incarceration in a diaphragmatic hernia. In most of the previously published cases, the herniation of the colon occurred in the left thorax. The main aetiology for diaphragmatic hernias is blunt abdominal trauma and penetrating injuries [1]. 

Bochdalek hernia is a congenital diaphragmatic defect that occurs in about 2,200 to 12,500 live births. As this type of defect is usually symptomatic and badly tolerated, diagnosis is usually made in neonates, and only 5% are discovered after childhood. Right-sided Bochdalek hernias are far less common than left-sided ones (80–90%) [2]. This is presumably due to the pleuroperitoneal canal closes earlier on the right side or to narrowing of the right pleuroperitoneal canal by the caudate lobe of the liver. Contrasting with this assumption and with the previously published data, a retrospective analysis of 13,138 abdominal CT reports found a higher incidence of right-sided hernias (68%) among asymptomatic adults (22 patients representing an incidence of 0.17%, with a female prevalence of 77%) [3]. 

Only one case of caecal perforation through a Bochdalek hernia has been reported in the literature [4]. In that case, the hernia was left-sided, with the transverse colon entering the posterolateral defect; caecal perforation resulted in peritonitis.

An extensive review of 14 cases right-sided Bochdalek hernias obstructing in adults revealed that 43% of the reported cases presented with acute abdominal pain, 36% with respiratory symptoms, and 27% with symptoms of obstruction [5]. In half of the cases, the hernia sac contained colon and multiple other viscera in 40%. In only four cases, only the small bowel was incarcerated, and signs of strangulation were evident in only one case [6–9]. In one case, the hernia included the ileum, colon, and gallbladder [10]. These five patients were aged from 32 to 74 years (three females, two males). The main clinical features were abdominal pain (three cases), intestinal obstruction (two cases), and respiratory symptoms (together with abdominal pain in one case). Three patients were treated by laparotomy, one by thoracotomy, and one by combined approach; they all survived. Other isolated case reports of right-sided Bochdalek hernias have been published. For a 38-year-old woman, the diagnosis was made after evidence of mild epigastric pain and minimal right pleural effusion on chest X-ray [11]. The right lobe of the liver, flexura hepatica of the colon, and omentum were herniated into the thorax cavity; the diaphragmatic defect was repaired by thoracotomy. For another 75-year-old woman admitted with cough and palpitation, herniation of the small and large bowels in a right posterior diaphragmatic (without ischemia) was successfully treated by thoracotomy [12].

Since the symptoms are varied, the diagnosis may be delayed, particularly when routine chest X-ray examination is not conclusive or is misinterpreted. Gastrointestinal symptoms related to the obstruction of the herniated organ are more commonly seen, but some patients may present symptoms such as cough, breathlessness, and palpitation but no major abdominal complaints. A 35-year-old woman typically presented with central abdominal pain, vomiting, and tenderness at the palpation of the right upper quadrant [13]. The chest X-ray revealed bowel loops under the right hemidiaphragm, above the liver. This was erroneously diagnosed as Chilaiditi's sign (hepatodiaphragmatic interposition of bowel in usually asymptomatic patients) [14]. The correct diagnosis was made 48 hours later, when the patient developed clinical signs of peritonitis. At laparotomy, the mid-jejunum was found to be incarcerated in a right-sided Bochdalek hernia, and ischemic small bowel was also found inside the abdominal cavity. The patient recovered following surgery. 

The initial chest X-ray may be also misinterpreted as tension pneumothorax or lung consolidation with empyema [15]. Faecal drainage from the chest tube is then highly indicative of perforation. 

Additionally, the detection of congenital diaphragmatic hernia may be missed because of intermittent herniation of the abdominal viscera into the thoracic cavity. Our patient had been investigated two years before by a thoracoabdominal CT when she was admitted to the hospital for a respiratory disease. Helical CT is extremely valuable in the detection of diaphragmatic injuries with an accuracy of 88% for left-sided injuries and 70% for right-sided injuries [16]. At that time, there was evidence of a small posterior diaphragmatic defect containing a very limited portion of the liver, but no immediate surgical repair was proposed. Herniation of the caecum must have occurred spontaneously during the past two years. The common factors associated to herniation (recent trauma, severe strain, and obesity) were not present.

Transabdominal and transthoracic approaches have been recommended for Bochdalek hernia repair [17]. When the patient has signs of intestinal obstruction or strangulation, as in our case, the abdominal approach with midline incision is the first option. A thoracotomy could be additionally required if the intrathoracic bowel cannot be reduced transdiaphragmatically. If there is no incarcerated bowel, transthoracic repair of a right diaphragmatic hernia seems more appropriate.

In conclusion, a rare case of right-sided Bochdalek hernia with intrathoracic caecal perforation is described. The poor prognosis is due to the delayed diagnosis and to the extensive infectious complications related to the chronic use of immunosuppressive drugs [18]. It is emphasized that with congenital diaphragmatic hernias the symptoms and signs of bowel perforation may also include respiratory distress and chest infections [2].
